# Identification of the high-risk population facing early death in older patients with primary intracranial glioma: a retrospective cohort study

**DOI:** 10.3389/fendo.2025.1546530

**Published:** 2025-03-03

**Authors:** Gui-Jun Lu, Ying Zhao, Rui Huang

**Affiliations:** ^1^ Department of Neurosurgery, the Second Hospital of Jilin University, Changchun, Jilin, China; ^2^ Department of Hand Surgery, the Second Hospital of Jilin University, Changchun, Jilin, China

**Keywords:** early death, high-risk, older, intracranial glioma, nomogram, SEER

## Abstract

**Background:**

This study aimed to establish a diagnostic nomogram to predict the early death risk in older patients with primary intracranial glioma and to identify the high-risk population in those patients to provide them with specialized care to increase their benefit from survival.

**Methods:**

Patients aged 60 years and older with histologically confirmed intracranial glioma were identified in the Surveillance, Epidemiology and End Results (SEER) database. Initially, they were divided into a training set and a validation set in a 7:3 ratio. Next, univariate and multivariate logistic regression were employed to identify independent risk variables, which were used to develop a diagnostic nomogram further. Additional analyses were performed on the diagnostic nomogram’s performance, including calibration curves, receiver operating characteristic (ROC) curves, and decision curve analysis (DCA). A mortality risk classification system was ultimately developed using the diagnostic nomogram.

**Results:**

This study included 8,859 individuals diagnosed with primary intracranial glioma. The participants were randomly split into two groups: a training set consisting of 6203 individuals and a validation set consisting of 2,656 individuals, with a ratio of 7 to 3. Univariate and multivariate logistic regression analyses on early death showed 7 independent risk variables (age, median household income, histological type, tumor grade, surgery, radiation therapy, and systemic therapy sequence with surgery) in the training set. A diagnostic nomogram for predicting the early death risk was created based on these variables. Calibration curves showed a high agreement between the expected and actual probabilities. The area under the curves (AUC) for the training and validation sets were 0.798 and 0.811, respectively. Meanwhile, the novel-created diagnostic nomogram had the highest AUC value compared to each independent risk variables, which showed that the nomogram had the best discriminatory ability. The DCA indicated that the nomogram has the potential to provide greater clinical advantages across a broad spectrum of threshold probabilities. Furthermore, a nomogram-based risk classification system was constructed to help us identify the high-risk population facing early death.

**Conclusions:**

This study created a novel diagnostic nomogram to predict the probability of early death in older patients with intracranial glioma. In the meantime, a nomogram-based risk classification system was also constructed to help us identify the high-risk population facing early death in older patients with intracranial glioma and provide them with specialized care to increase their benefit from survival.

## 1 Introduction

According to the latest findings on cancer data, it is estimated that there will be 18,760 deaths and 25,460 new cases of brain and other nervous system cancers in the United States in 2024 (1). Gliomas, which arise from glial cells in the central nervous system, are the predominant type of intracranial tumors. Malignant glioma poses numerous risks, such as its elevated malignancy, mortality rate, and likelihood of recurrence. The impact on society and families has been significant. According to data from the Central Brain Tumor Registry of the United States (CBTRUS), the typical yearly age-adjusted occurrence rate of all malignant brain and other CNS tumors was 7.02 per 100,000 individuals, with a typical yearly death rate of 4.41 per 100,000 individuals (2). Furthermore, the frequency and death rate of intracranial glioma rise notably as individuals get older. The five-year relative survival rate of malignant brain tumors and other CNS tumors is 35.7%, but for those over 40 years old, it is only 21.0%.

With the continuous development and advancement of diagnostic and therapeutic techniques, surgery combined with systemic therapy (radiotherapy and chemotherapy) has dramatically improved the survival benefit of patients with intracranial gliomas (3). However, the prognosis for the older population remains poor (4). According to the population data of 2019-2020 available from the Surveillance, Epidemiology, and End Results (SEER) database, there were 16,653 new patients with intracranial tumors, with 53.2% of the population over 60 years old. And 35.16% of those individuals had a survival time of just less than 6 months. Such a high early death rate has become a significant challenge for the survival benefit of patients.

In the face of such a severe situation, how to quickly identify patients at high risk of early death and provide effective interventions to maximize the therapeutic benefit has become an urgent challenge for neurosurgery surgeons (5). A nomogram is a visual computational tool used to predict disease outcomes, thereby improving the accuracy of cancer prognosis prediction, which has been proven to be superior to TNM staging (6, 7). Nomograms offer the benefit of personalized risk assessment, aiding doctors in creating tailored treatment plans for individual patients (6). Additionally, they assist in creating a risk classification system, which helps identify high-risk groups and allows for the rational allocation of healthcare resources by doctors. Currently, there are nomograms for predicting overall survival or cancer special survival in glioma patients, and there are epidemiologic studies of glioma patients of different ages (8, 9). Still, there are no studies to construct nomograms for predicting early death in older patients with intracranial gliomas.

Hence, a diagnostic nomogram was created and confirmed in this study to estimate the likelihood of early death in older patients with primary intracranial glioma using information from the SEER database. Then, risk classification of older patients with primary intracranial glioma is realized by the nomogram. Then, subgroup analysis is performed to screen out the high-risk populations facing early death and give more effective treatment at the initial diagnoses to achieve the purpose of better patient management and rational allocation of healthcare resources.

## 2 Methods

### 2.1 Selection of patients

This study included patients who were diagnosed with primary intracranial glioma in the SEER database. The SEER database is a public database that includes tumor data from 28% of the U.S. population, covering a wide range of data on patient demographics, primary tumor sites, treatment information, and survival rates. Notably, it avoids the potentially limiting bias that arises when evaluating patients from a single institution by allowing the use of data from multiple centers of patients. Using the SEER*Stat software version 8.4.3 from www.seer.cancer.gov, we successfully obtained patient data from [Incidence-SEER Research Date, 17 Registries, Nov 2022 Sub (2000–2020)]. Since the SEER database is accessible to researchers worldwide, our study does not necessitate approval from an ethical committee or consent from patients.

**Figure 1:**
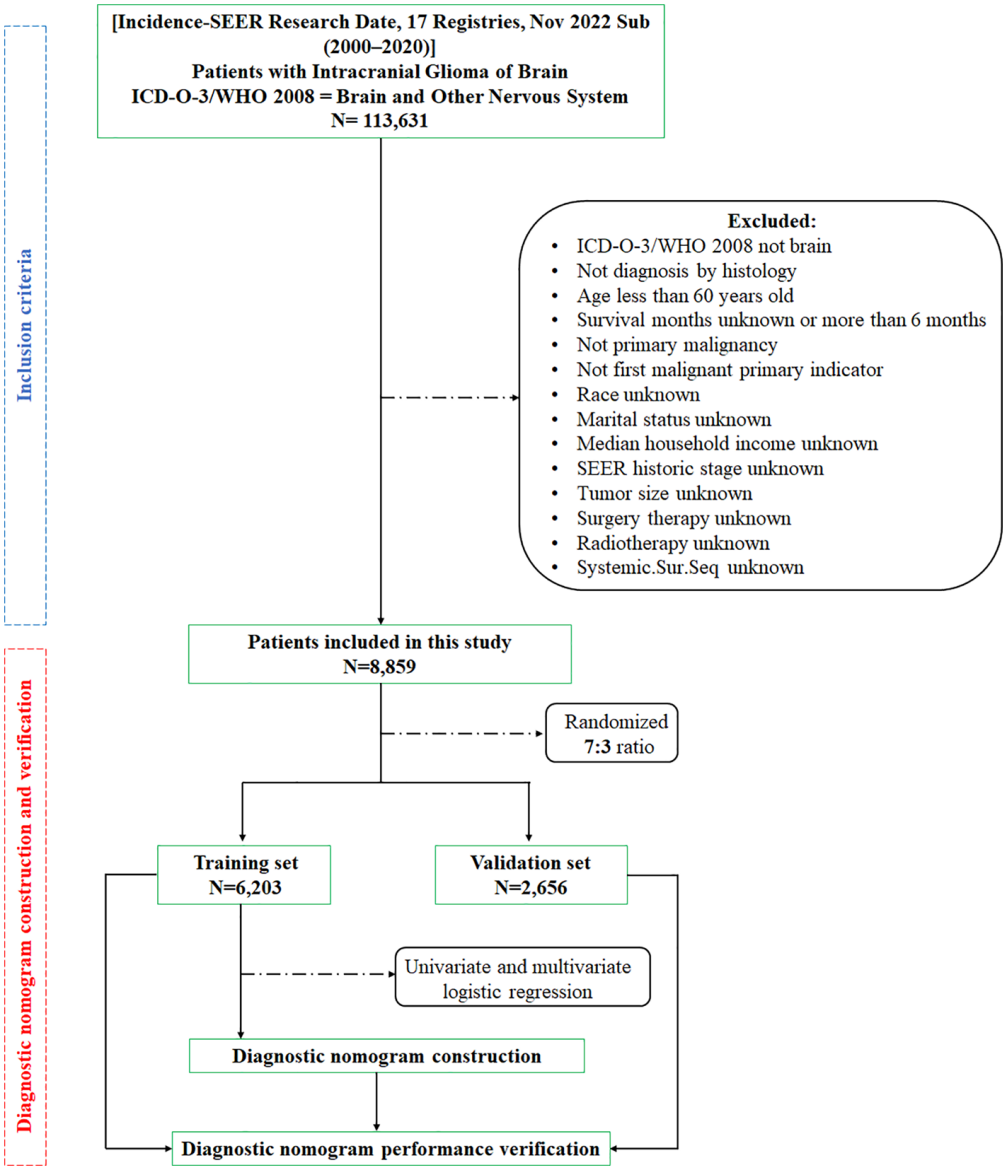
The study’s patient selection and workflow.

These were the inclusion criteria: (1) tumors located in the brain; (2) age≥60 years old; (3) the survival period was between one month and six months; (4) intracranial glioma is the initial primary tumor; and (5) diagnosed by histology. Meanwhile, the exclusion criteria were as follows: (1) ICD-O-3/WHO 2008 not brain; (2) not primary malignancy; (3) the survival period was unknown, or more than six months; (4) age less than 60 years old. The participants were divided into a training set and a validation set in a 7:3 ratio through random selection (10, 11). **Figure 1** displays the process of selecting patients for this study.

### 2.2 Definition of variables

Early death in this study was identified to be death within 6 months from initial diagnosis (12, 13). The older patient in this study was identified as a patient who was no less than 60 years old at the initial diagnosis (14–16).

Our study initially included 19 variables that might relate to early death in older patients with primary intracranial glioma. The X-tile program identifies the best thresholds for age and tumor size, which were found to be 72 years and 79 years old, and 26 mm and 66 mm, respectively (17). Age categories included 60-71, 72-79, and over 79 years old, while tumor size categories were less than 26, 26-66, and greater than 66 mm. Gender was categorized as either male or female. The classification of race included categories for black, white, and other ethnicities such as American Indian/AK Native and Asian/Pacific Islander. Marital status was categorized as either married or unmarried, and unmarried included single, unmarried, domestic partner, widowed, separated, and divorced. Median household income was divided into <$50,000, $50,000-74,999, and >$74,999. Laterality was categorized as left, right, bilateral, or other. The tumor stage was divided into localized, regional, and distant. Tumor grade was divided into grades I-II, III-IV, and unknown. The different histologic categories included glioblastoma, non-glioblastoma astrocytoma, oligodendroglial tumors, ependymoma, and unspecified gliomas, as per the International Classification of Diseases for Oncology, Third Edition (ICD-O-3) codes (18). Metastases to distant sites such as bone, brain, liver, and lung were categorized as either present, absent, or unknown. Treatment (surgery, radiotherapy, chemotherapy) was divided into yes or no. Radiation sequence with surgery was divided into no radiation and/or cancer-directed surgery, radiation after surgery, and other. Systemic therapy sequence with surgery was divided into no systemic therapy and/or surgical procedures, systemic therapy after surgery, and other.

### 2.3 Statistical analysis

Using R software, the patients were divided into training and validation sets in a 7:3 ratio through random selection. The training set was used to determine independent risk variables associated with early death in older patients with primary intracranial glioma and to develop a diagnostic nomogram, which was confirmed by the validation set later. Univariate logistic regression analysis was performed to determine the relationship between the included variables and early death in patients, showing hazard ratios (HR) and 95% confidence intervals (CI) to demonstrate the impact of these variables on early death. To determine early death-related independent risk variables, multivariate logistic regression analysis further explored variables with a *p* < 0.05. A diagnostic nomogram was created to predict early death in older patients with primary intracranial glioma based on these independent risk variables. Meanwhile, each independent prognostic variable obtained a special point. A calibration curve was then used to demonstrate the validity of the diagnostic nomogram after the nomogram was constructed. The area under the curve (AUC) was determined by analyzing ROC curves to assess the diagnostic nomogram’s ability to discriminate. The ROC curves of each independent risk variable and the diagnostic nomogram were generated to illustrate their predictive capabilities using the provided AUC value, where a higher AUC indicates stronger predictive ability. Decision curve analysis (DCA) assessed the nomogram’s clinical utility.

A classification system was developed using the nomogram to help determine the mortality risk for these patients. According to the nomogram, the patient’s total points were calculated, and cut-off values for early death risk were identified using X-tile software. Subsequently, a mortality risk classification system for classifying mortality risk was created to categorize these individuals into subgroups with low and high risks of early death. We analyzed the Kaplan-Meier survival curves to determine if there were any disparities between the two risk categories. This study utilized SPSS (version 27.0) and R (version 4.1.0) for statistical analysis, with significance determined at *p*< 0.05.

## 3 Results

### 3.1 Demographic and clinicopathologic characteristics

This study included a total of 8,859 older individuals with primary intracranial glioma diagnosed between 2000 and 2020, based on the specified criteria for inclusion and exclusion. Participants were divided into two groups, a training set consisting of 6,203 individuals and a validation set consisting of 2,656 individuals, with a ratio of 7 to 3. The patient’s detailed demographic information, tumor characteristics, and treatment were summarized in **Table 1**.

**Table 1:** Patient demographics and baseline characteristics.

Characteristics	Training set (n=6,203)		Validation set (n=2,656)	
	Alive	Dead	Alive	Dead
	427	5,776	215	2,441
Age (years)				
60-71	270	2,863	152	1,192
72-79	128	1,854	51	738
>79	29	1,059	12	511
Sex				
Female	177	2,691	88	1,102
Male	250	3,085	127	1,339
Race				
Black	26	265	10	114
White	375	5,277	187	2,219
Other	26	234	18	108
Marital status				
No	143	2,235	66	892
Yes	284	3,541	149	1,549
Median household income				
<$50,000	27	673	19	295
$50,000-$74,999	132	2,880	59	1,214
>$74,999	268	2,223	137	932
Laterality				
Left	170	2,353	97	1,007
Right	202	2,440	100	1,011
Bilateral	5	109	2	48
Other	50	874	16	375
Tumor size (mm)				
<26	68	796	38	340
26-66	311	4,346	158	1,841
>66	48	634	19	260
Tumor grade				
Grade I-II	12	41	1	18
Grade III-IV	166	2,199	73	961
Unknown	249	3,536	141	1,462
Tumor stage				
Localized	356	4,446	182	1,855
Regional	69	1,198	31	529
Distant	2	132	2	57
Histological type				
Glioblastoma	274	5,096	197	2,164
Non-glioblastoma astrocytoma	27	498	13	205
Oligodendroglial tumors	13	86	2	33
Ependymoma	6	15	2	6
Glioma, NOS	7	81	1	33
Bone metastasis				
No	411	3,733	211	1,558
Yes	0	4	0	2
Unknown	16	2,039	4	851
Brain metastasis				
No	409	3,689	210	1,574
Yes	2	47	1	18
Unknown	16	2,040	4	849
Liver metastasis				
No	411	3,734	211	1,590
Yes	0	3	0	0
Unknown	16	2,039	4	851
Lung metastasis				
No	411	3,735	211	1,585
Yes	0	3	0	4
Unknown	16	2,038	4	852
Surgery				
No	54	1,807	22	774
Yes	373	3,969	193	1,667
Radiation therapy				
No	71	2,339	31	982
Yes	356	3,437	184	1,459
Chemotherapy				
No	101	2,993	35	1,265
Yes	326	2,783	180	1,176
Surg.Rad.Seq				
No radiation and/or cancer-directed surgery	113	3,378	48	1,430
Radiation after surgery	311	2,372	166	999
Other	3	26	1	12
Systemic.Sur.Seq				
No systemic therapy and/or surgical procedures	125	3,090	50	1,292
Systemic therapy after surgery	292	1,658	161	736
Other	10	1,028	4	413

Surg.Rad.Seq: Radiation sequence with surgery.

Systemic.Sur.Seq: Systemic therapy sequence with surgery.

### 3.2 Identification of independent risk variables

First, the 19 variables in the training set were enrolled in univariate logistic regression analysis. The findings indicated that 16 variables were associated with the occurrence of early death in older individuals diagnosed with primary intracranial glioma, with a *p*-value less than 0.05, excluding race, laterality, and tumor size, which had a p-value greater than 0.05. Next, those 16 variables were included in a multivariate logistic regression analysis to remove the effects of confounding variables. The findings indicated that variables such as age, median household income, histological type, tumor grade, surgery, radiation therapy, and systemic therapy sequence with surgery were identified as independent risk variables of early death in older individuals with primary intracranial glioma, while variables like gender, marital status, tumor stage, distant metastasis to bones, brain, liver, or lung, chemotherapy, and radiation sequence with surgery did not show statistical significance (*p* > 0.05, **Table 2**).

**Table 2:** Analyzing the risk factors for early death in older individuals with primary malignant intracranial glioma using both univariate and multivariate logistic regression.

Characteristics	Univariate analysis		Multivariate analysis	
	HR (95%CI)	P	HR (95%CI)	P
Age (years)				
60-71	Reference		Reference	
72-79	1.37(1.1-1.7)	0.005	1.18(0.94-1.49)	0.16
>79	3.44(2.33-5.08)	<0.001	2.42(1.61-3.64)	<0.001
Sex				
Female	Reference		Reference	
Male	0.81(0.66-0.99)	0.04	0.94(0.75-1.16)	0.552
Race				
Black	Reference			
White	1.38(0.91-2.09)	0.129		
Other	0.88(0.5-1.56)	0.67		
Marital status				
No	Reference		Reference	
Yes	0.8(0.65-0.98)	0.033	0.94(0.75-1.18)	0.619
Median household income				
<$50,000	Reference		Reference	
$50,000-$74,999	0.88(0.57-1.34)	0.537	0.81(0.53-1.26)	0.354
>$74,999	0.33(0.22-0.5)	<0.001	0.29(0.19-0.44)	<0.001
Laterality				
Left	Reference			
Right	0.87(0.71-1.08)	0.207		
Bilateral	1.58(0.63-3.91)	0.328		
Other	1.26(0.91-1.75)	0.159		
Tumor size (mm)				
<26	Reference			
26-66	1.19(0.91-1.57)	0.204		
>66	1.13(0.77-1.66)	0.538		
Tumor grade				
Grade I-II				
Grade III-IV	3.88(2-7.52)	<0.001	5.13(2.21-11.89)	<0.001
Unknown	4.16(2.16-8.01)	<0.001	6.72(2.88-15.67)	<0.001
Tumor stage				
Localized	Reference		Reference	
Regional	1.39(1.07-1.81)	0.015	1.27(0.96-1.69)	0.095
Distant	5.28(1.3-21.43)	0.02	17.38(0.72-419.31)	0.079
Histological type				
Glioblastoma	Reference		Reference	
Non-glioblastoma astrocytoma	1.35(0.91-2.02)	0.139	1.09(0.69-1.7)	0.722
Oligodendroglial tumors	0.49(0.27-0.88)	0.017	0.36(0.18-0.7)	0.003
Ependymoma	0.18(0.07-0.48)	<0.001	0.08(0.03-0.25)	<0.001
Glioma, NOS	0.85(0.39-1.85)	0.681	0.6(0.26-1.38)	0.227
Bone metastasis				
No	Reference		Reference	
Yes	85792.96(0-6.4424289240477e+232)	0.966	15619.07(0-Inf)	0.981
Unknown	14.03(8.49-23.19)	<0.001	10.25(0-22475.26)	0.553
Brain metastasis				
No	Reference		Reference	
Yes	2.61(0.63-10.77)	0.186	0.17(0.01-4.18)	0.277
Unknown	14.14(8.55-23.36)	<0.001	5.8(0.05-679.45)	0.469
Liver metastasis				
No	Reference		Reference	
Yes	85769.98(0-1.15188668792783e+268)	0.971	6159(0-Inf)	0.985
Unknown	14.03(8.49-23.18)	<0.001	0.54(0-56083.49)	0.916
Lung metastasis				
No	Reference		Reference	
Yes	85747.02(0-1.15157828582012e+268)	0.971	7718(0-Inf)	0.985
Unknown	14.02(8.48-23.16)	<0.001	0.34(0-9733.54)	0.838
Surgery				
No	Reference		Reference	
Yes	0.32(0.24-0.43)	<0.001	0.47(0.25-0.89)	0.02
Radiation therapy				
No	Reference		Reference	
Yes	0.29(0.23-0.38)	<0.001	0.33(0.15-0.71)	0.005
Chemotherapy				
No	Reference		Reference	
Yes	0.29(0.23-0.36)	<0.001	1.05(0.61-1.8)	0.867
Surg.Rad.Seq				
No radiation and/or cancer-directed surgery	Reference		Reference	
Radiation after surgery	0.26(0.2-0.32)	<0.001	1.6(0.7-3.67)	0.267
Other	0.29(0.09-0.97)	0.045	1.3(0.29-5.8)	0.733
Systemic.Sur.Seq				
No systemic therapy and/or surgical procedures	Reference		Reference	
Systemic therapy after surgery	0.23(0.18-0.29)	<0.001	0.38(0.2-0.7)	0.002
Other	4.16(2.18-7.95)	<0.001	1.04(0.46-2.37)	0.923

Surg.Rad.Seq: Radiation sequence with surgery.

Systemic.Sur.Seq: Systemic therapy sequence with surgery.

### 3.3 Construction and validation of the diagnostic nomogram

**Figure 2:**
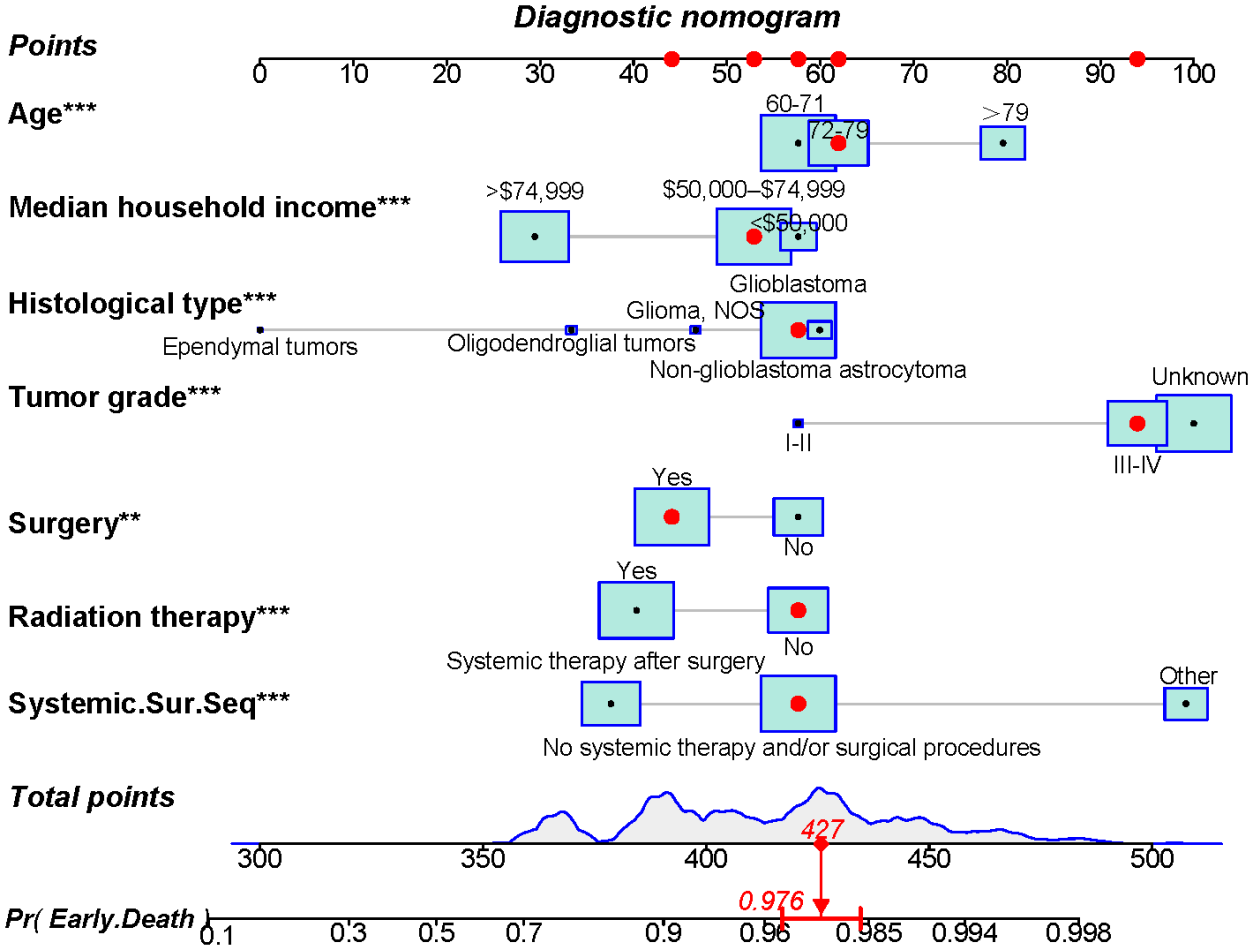
A novel diagnostic nomogram for forecasting the likelihood of early death in older individuals with primary intracranial glioma. **p < 0.05, ***p < 0.01.

**Figure 3:**
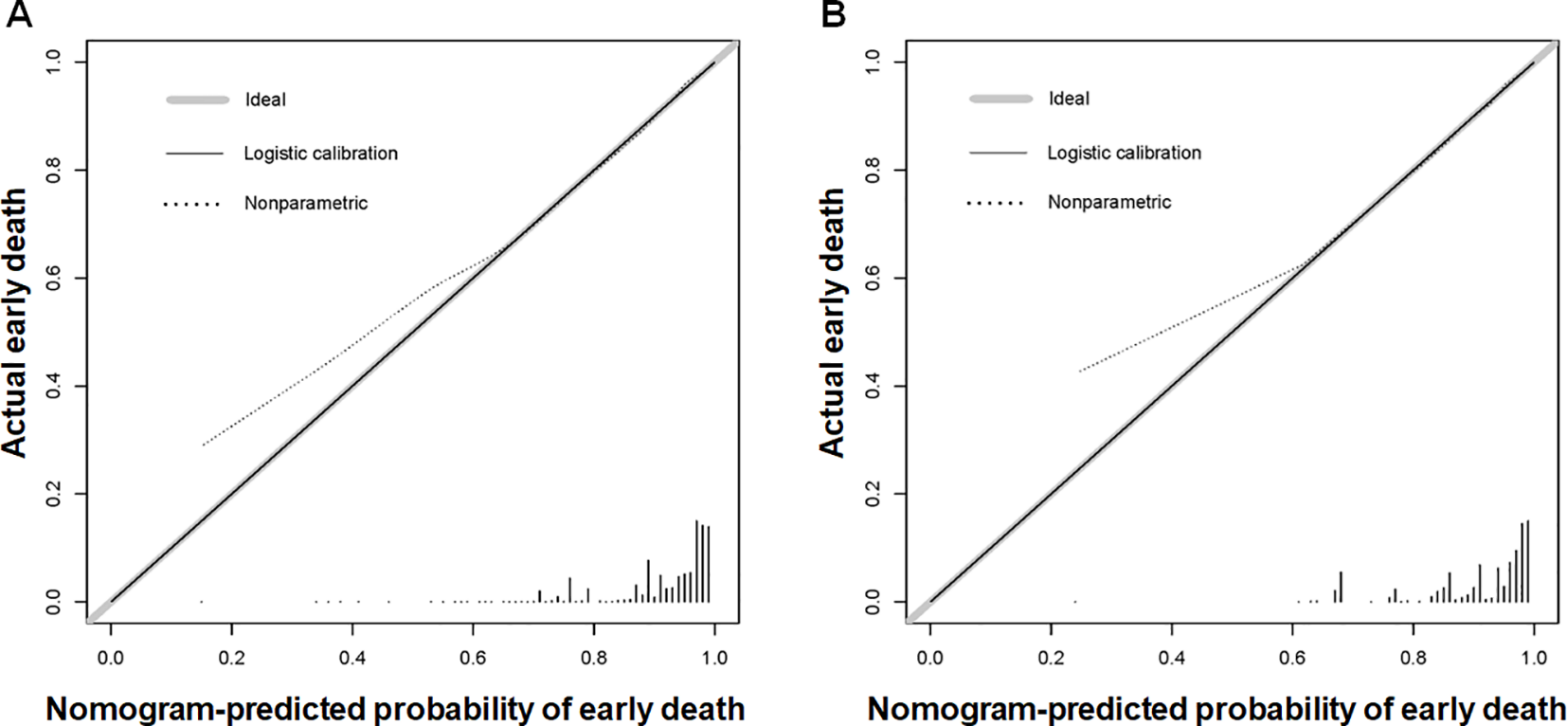
Calibration curves for the diagnostic nomogram were generated for both the training **(A)** and validation **(B)** sets. The Y-axis shows the actual chance of an early death in older patients with primary intracranial glioma, whereas the X-axis shows the anticipated likelihood of the diagnostic nomogram.

**Figure 4:**
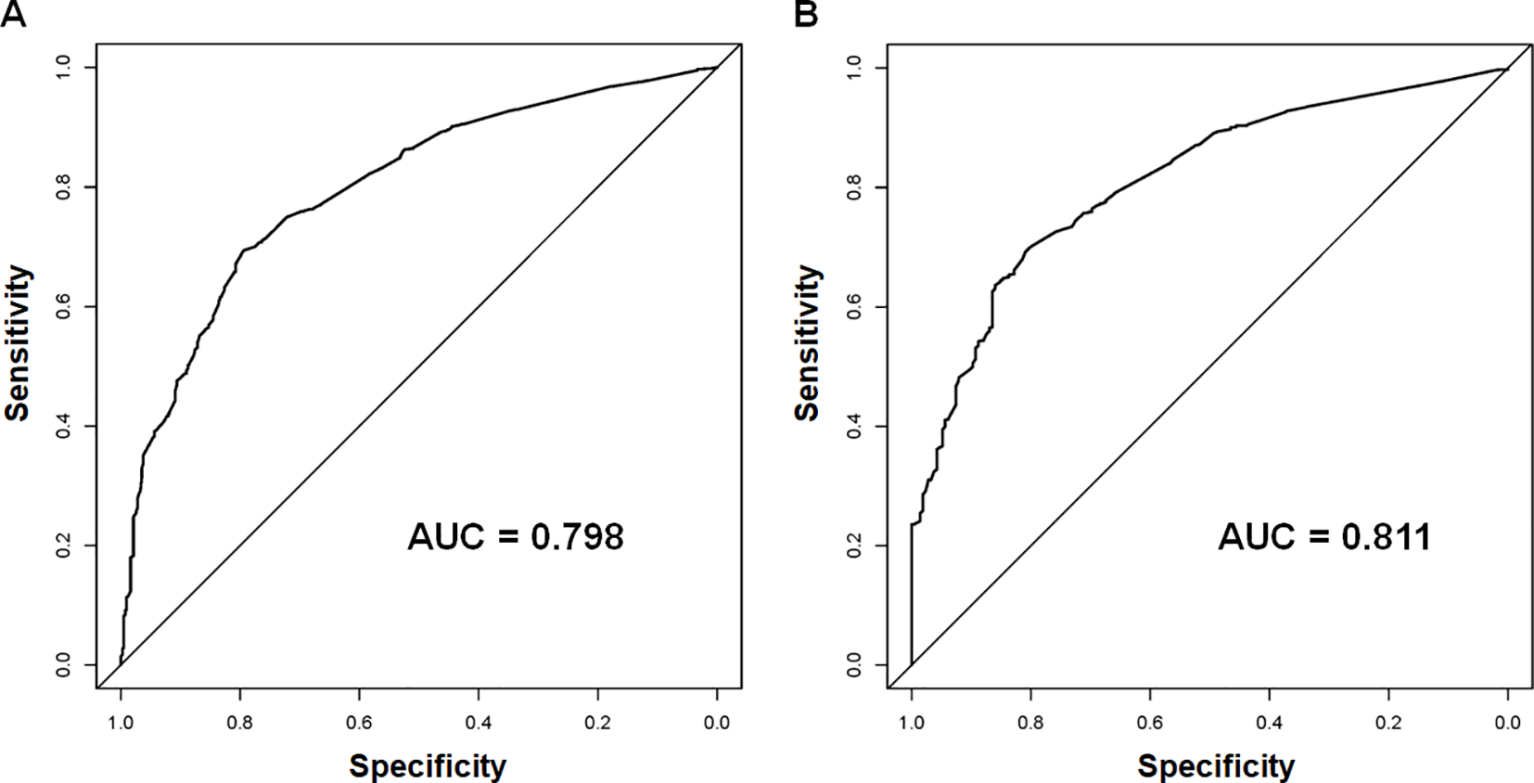
The receiver operating characteristic (ROC) curves and area under the curve (AUC) of the diagnostic nomogram were used to predict the early death of older patients with primary intracranial glioma in both training **(A)** and validation sets **(B)**.

**Figure 5:**
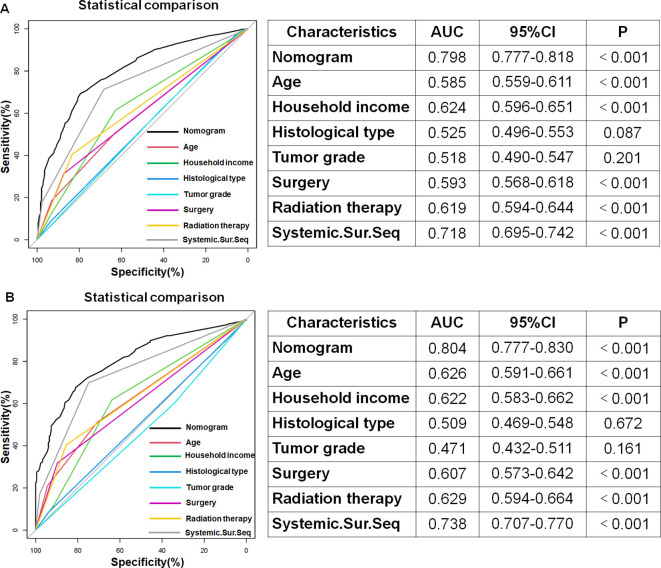
In both the training **(A)** and validation **(B)** sets, the diagnostic nomogram had the highest AUC compared to each independent risk variable, demonstrating its outstanding predictive ability in estimating the probability of early death in older patients with primary intracranial glioma.

**Figure 6:**
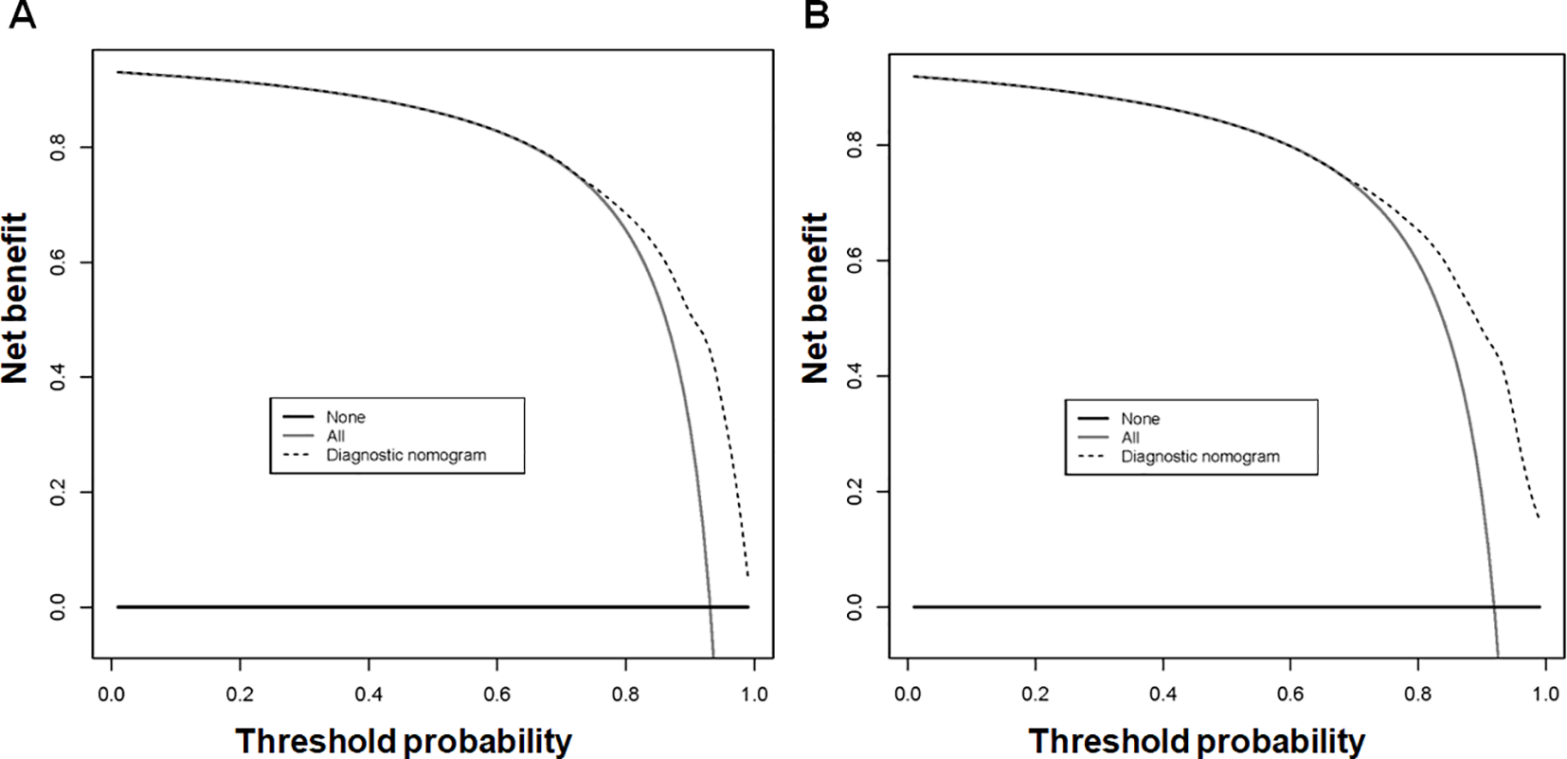
The decision curve analysis (DCA) of the diagnostic nomogram was utilized to predict early death in older individuals with primary intracranial glioma in both training **(A)** and validation sets **(B)**.

Using the 7 independent risk variables mentioned earlier, a diagnostic nomogram was developed in R to predict the early death in older patients with primary intracranial glioma. Additionally, each independent risk variable was assigned a specific point value in the nomogram based on its impact on the outcome, as detailed in **Table 3**. The total point of a patient was obtained by summing the point of each independent risk variable. To determine the likelihood of early death in these individuals, one can draw a vertical line from the total points to the axis representing early death (**Figure 2**). The calibration curves for the training and validation sets closely matched the ideal 45°curve (**Figure 3**), suggesting that the predicted early death rates from our diagnostic nomogram were in strong agreement with the actual ones, confirming the high predictive accuracy of the diagnostic nomogram. **Figure 4** displays the ROC curves for both the training and validation sets. The diagnostic nomogram showed high discrimination with AUCs of 0.798 and 0.811 for the training and validation sets, respectively. The diagnostic nomogram showed higher AUCs compared to each independent risk variable in both sets, indicating strong prediction accuracy (**Figure 5**). Additionally, DCA demonstrated a positive clinical benefit net within a specific threshold range in both sets, suggesting the promising future clinical effectiveness of the nomogram (**Figure 6**).

**Table 3:** The detailed point of each early death-related independent risk factor in this diagnostic nomogram.

Variables	Corresponding point
Age (years)	
60-71	58
72-79	62
>79	80
Median household income	
<$50,000	58
$50,000-$74,999	53
>$74,999	29
Tumor grade	
Grade I-II	58
Grade III-IV	94
Unknown	100
Histological type	
Glioblastoma	58
Non-glioblastoma astrocytoma	60
Oligodendroglial tumors	33
Ependymoma	0
Glioma, NOS	47
Surgery	
No	58
Yes	44
Radiation therapy	
No	58
Yes	40
Systemic.Sur.Seq	
No systemic therapy and/or surgical procedures	58
Systemic therapy after surgery	38
Other	99

Systemic.Sur.Seq: Systemic therapy sequence with surgery.

### 3.4 Construction of the early death risk classification system

**Figure 7:**
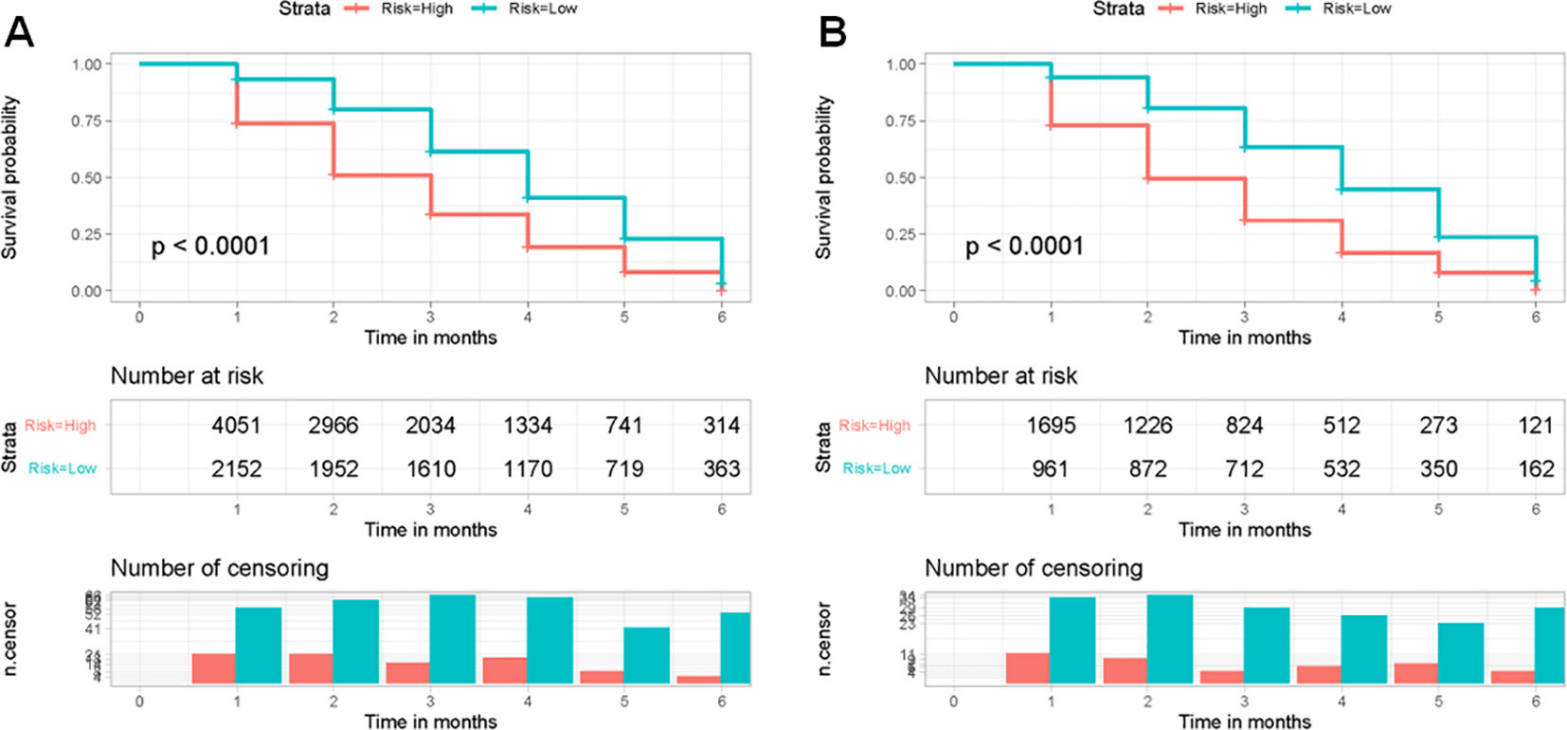
Kaplan-Meier survival curves were used to analyze early death in two different risk categories of older individuals with primary intracranial glioma in both the training **(A)** and validation sets **(B)**.

The X-tiles software was used to determine the optimal threshold of 400 based on the total points of each patient in the training set. Patients were categorized into two groups based on risk level: low-risk (≤400) and high-risk (>400). Next, the Kaplan-Meier survival curves for the two risk categories were created and the log-rank test was conducted. **Figure 7** displayed a notable distinction between the two subgroups (*p* < 0.05) in the training and validation sets, confirming the effectiveness of the risk classification system based on the nomogram. Therefore, the nomogram could help us to identify the high-risk population facing early death in older patients with intracranial glioma.

## 4 Discussion

Glioma, with multiple subtypes, is the predominant form of malignant primary central nervous system tumor (19). Compared with other tumors, the incidence of glioma is relatively rare, but its progression occurs rapidly with a low survival rate (20). The progression of intracranial glioma is always influenced by a variety of factors, including age, sex, tumor grade, histological type, surgical resection, radiation, chemotherapy, and so on (21). And many patients with gliomas would end up with unavoidable death due to tumor recurrence and metastasis. Clinical management of older patients with primary intracranial glioma is challenging (22). At the same time, excessively high early death rates constrain the therapeutic benefit to the population, causing not only great psychological trauma to patients but also a significant increase in the burden of disease on society as aging societies intensify globally. Therefore, more attention should be devoted to decreasing the early death rate in those populations.

In clinical practice, it is difficult to predict the early death of a patient based on a single variable. It is necessary to combine several or even more than a dozen variables closely related to the prognosis to improve the accuracy of the prediction (5). The diagnostic accuracy of traditional staging systems, such as the AJCC TNM staging, which classifies patients into four stages based on the all-or-nothing principle of variable categorization, has long been surpassed by emerging predictive models, such as nomogram, machine learning, and so on. The nomogram has been widely used in medical research as a simple graphical representation that provides an individualized prediction of survival outcomes and has become an essential component of modern medical decision-making. It contains multiple prognostic variables, including continuous variables, and describes factors and outcomes associated with more complex relationships toward prognosis. Thus, a novel diagnostic nomogram was created in this research to forecast early death in older patients with primary intracranial glioma using both univariate and multivariate logistic regression analyses, aiming to offer personalized early death evaluation and risk categorization. Our study found that age at diagnosis, median household income, histological type, tumor grade, surgery, radiation therapy, and systemic therapy sequence with surgery were identified as independent risk variables by analyzing data from 8,859 older patients in the SEER database. A diagnostic nomogram was created and validated based on those independent risk variables to predict early death in older patients with intracranial glioma. The calibration curves in both the training and validation sets closely resembled the 45° ideal curve, suggesting a strong agreement between the expected and observed probabilities. The diagnostic nomogram had higher AUCs compared to each independent risk variable in both sets, indicating its strong predictive accuracy. Additionally, DCA demonstrated an excellent positive clinical benefit net within a specific threshold range in both sets, suggesting the promising future clinical effectiveness of the nomogram. Moreover, utilizing a risk stratification system based on nomograms could classify patients into low-risk and high-risk categories, enabling the identification of older patients with intracranial glioma who are at high risk of early death.

Numerous research studies have indicated that advancing age is a negative predictive element for individuals diagnosed with primary intracranial glioma (23, 24). Additionally, our research indicated that advancing age is an independent risk variable to early death. On the one hand, it may be attributed to the poorer overall health of older patients as they face more preoperative complications such as hypertension and diabetes, which challenge surgical tolerance and some other postoperative complications (25). Hence, their acceptance of surgical treatment is considerably lower, with 72.93% and 61.82% for 60-71 and > 79-year-old patients, respectively. On the other hand, a dysregulated immune response may contribute to the poorer prognosis of the older. Patients’ ability to inhibit tumor growth and delay distant metastatic spread decreases as they age. Moreover, median household income is also an independent risk variable. More household income guarantees access to better healthcare resources, and they also tend to have a more positive attitude towards treatment, both in terms of treatment of the disease itself and nutritional intake, than those with lower household incomes. Therefore, the higher the income, the lower the possibility of early death.

Furthermore, early death was associated with the histological type and tumor grade. The most prevalent histological types of intracranial glioma were glioblastoma and non-glioblastoma astrocytoma, both classified as high-grade glioma along with Grade III and IV. Meanwhile, oligodendroglioma and astrocytoma were defined as low-grade glioma with Grade I and II (4, 18, 21). Low-grade gliomas can be easily removed and are well distinguished based on the location of tumor development, leading to a more favorable outcome compared to high-grade gliomas, which are mainly undifferentiated tumors with a grim prognosis (26–28).

Currently, intracranial gliomas are mainly treated with surgical resection, radiotherapy, and chemotherapy. Among them, surgery is the mainstay of treatment for all gliomas (22). Moreover, with the continuous development of neurosurgical techniques, surgical treatment of gliomas is safer than before (29). Compared with those treated with watch-and-wait in low-grade intracranial gliomas, patients who received early surgery benefit more survival time (30). Postoperative adjuvant radiotherapy is a critical element in the treatment plan for patients with primary intracranial glioma. It is usually recommended to start external radiation therapy within 2-4 weeks after surgical resection or biopsy.

Numerous research studies have indicated that the use of radiotherapy plays a crucial role in determining the prognosis for individuals with intracranial glioma and enhances their chances of survival (31–33). Sun et al.’s study showed the highest estimated hazard ratios for patients older than 60 years of age who did not receive radiotherapy. They also suggested that postoperative radiotherapy should be prioritized as part of the treatment plan for older patients (28). We reached similar conclusions in the present study.

Although the univariate logistic regression analyses proved the positive effect of chemotherapy in reducing early death, the multivariate regression analyses showed that chemotherapy was not an independent risk variable. However, the combination of postoperative radiotherapy and chemotherapy after surgery was an independent risk variable against early death, which is thought to coincide with the current treatment strategy for intracranial gliomas. The recommendations are inclined towards suggesting a combination of surgery, radiotherapy, and chemotherapy for more effective management of risks (3). Currently, the typical treatment for glioblastomas involves extensive surgery followed by radiotherapy along with additional temozolomide (TMZ) therapy. Since 2005, TMZ has been widely used to treat glioblastoma, and the survival rates of patients have improved, with a median survival rate of 14.6 months, a progression-free survival rate of 6.9 months, and a two-year overall survival rate of 26.5% (34–38). In addition, Li et al. demonstrated that the use of both radiotherapy and chemotherapy can decrease the possibility of death, regardless of whether there is an isocitrate dehydrogenase (IDH) gene mutation (3).

The advantages of this study are as follows. First, this study enrolled 8,859 candidates from 113,631 patients, and the results obtained are of good representative and clinical guidance value. Furthermore, the developed diagnostic nomogram demonstrates outstanding accuracy in predicting the likelihood of early death by tailoring the results to individual patients’ medical history. Finally, the mortality risk stratification system can help us identify the high-risk population and then realize the personalized management of patients and rational allocation of medical resources. Nevertheless, certain limitations in this research need to be acknowledged. First, selection bias will inevitably exist in any retrospective study. Furthermore, previous SEER databases did not contain data on certain molecular markers, like the 2016 World Health Organization’s categorization of CNS tumors, which utilized IDH 1/2 mutations and 1p/19q deletions for glioma classification (23, 24). Additional external research is necessary to confirm the effectiveness of the diagnostic nomogram in identifying the high risk of early death in older individuals with primary intracranial gliomas.

## 5 Conclusion

Overall, age, median household income, histological type, tumor grade, surgery, radiation therapy, and systemic therapy sequence with surgery were determined as independent risk variables for predicting early death in older individuals diagnosed with intracranial glioma. Utilizing these risk factors, we successfully designed and rigorously validated a new diagnostic nomogram. In the meantime, a nomogram-based risk categorization system that can distinguish between patients at high and low risk was created, which could help us to identify the high-risk population facing early death in older patients with intracranial glioma and provide them with specialized care to increase their benefit from survival.

## Data Availability

The original contributions presented in the study are included in the article/supplementary material. Further inquiries can be directed to the corresponding author.
